# New National Quarantine Law

**Published:** 1906-07

**Authors:** 


### Abstracts and Selections

### New National Quarantine Law.

*An Act to further protect the public health and make more effective the national quarantine.*

*Be it enacted by the Senate and House of Representatives of the United States of America in Congress assembled,* That the Secretary of the Treasury shall have the control, direction, and management of all quarantine stations, grounds, and anchorages established by authority of the United States, and as soon as practicable after the approval of this act shall select and designate such suitable places for them and establish the same at such points on or near the coast line of the United States or the border of the United States and a foreign country, as in his judgment are best suited for the same and necessary to prevent the introduction of yellow fever into the United States, and, in his discretion, he may also establish at the group of islands known as the Dry Tortugas, at the western end of the Florida reef, and at such other point or points on or near the coast line of the United States (not to exceed four in the aggregate) as he deems necessary, quarantine grounds, stations, and anchorages, whereat or whereto infected vessels bound for any port in the United States may be detained or sent for the purpose of being disinfected, having their cargoes disinfected and discharged, if necessary, and their sick treated in hospitals until all danger of infection or contagion from such vessels, their cargoes, passengers, or crews has been removed.

SEC. 2. That in cases in which the title to the land and water so selected and designated is in the United States it shall be the duty of the department, bureau, or official of the United States having custody or possession of such land and water, or any part thereof, not used by the Government for other purposes designated by law, or possession of said Dry Tortugas Islands, on demand of the Secretary of the Treasury, to deliver the same into his custody and possession for the use of the Public Health and Marine Hospital Service, evidencing such delivery by a suitable instrument in writing to be delivered to the Secretary of the Treasury. That in cases in which the title to such land and water, or any part thereof, is in any other owner than the United States it shall be the duty of the Secretary of the Treasury to secure the title and possession of the same to the United States for the use of the Public Health and Marine Hospital Service of the United States, by purchase at a reasonable price, if possible; but if, in his judgment, the price demanded for such property be excessive, he is hereby authorized to apply to the Attorney General of the United States to cause to be instituted, in the proper tribunal, condemnation proceedings in the name of the United States for the purpose of acquiring for the United States the title and possession of such land and water, and said Attorney General shall, as soon as possible after such application by the Secretary of the Treasury, cause such proceedings to be instituted and conducted to a conclusion, and the custody and possession of such land and water, when duly acquired in accordance with the award made in such condemnation proceedings, shall be delivered to the Secretary of the Treasury for the use of the Public Health and Marine Hospital Service.

SEC. 3. That on acquiring possession of any land and water in accordance with the provisions of this act for the purpose of establishing thereat a quarantine station and anchorage, the Secretary of the Treasury shall cause to be published in such newspapers as he may think proper, once a week for four successive weeks, a notice of the selection and designation of such places for quarantine stations and anchorages, with a description of the boundaries of such quarantine stations and anchorages, and such rules and regulations as he shall adopt and promulgate, requiring vessels with yellow fever among their passengers or crews to go to specified quarantine stations and anchorages, to be dealt with there before visiting any port of the United States. He shall establish at such quarantine stations and anchorages all necessary instrumentalities for disinfecting vessels and their cargoes, and where the same shall be required shall erect the necessary hospital buildings and install the necessary furniture and fittings for receiving and treating the sick among the passengers and crews of vessels going to such quarantine stations and anchorages, and provide for the separation of those among their passengers and crews who are suffering from yellow fever from those who are in good health, and shall further provide for doing all things necessary to eradicate such disease from such vessels, their cargoes, passengers, and crews.

SEC. 4. That any vessel, or any officer of any vessel, or other person other than State health or quarantine officers, entering within the limits of any quarantine grounds and anchorages, or any quarantine station and anchorage, or departing therefrom, in disregard of the quarantine rules and regulations or without the permission of the officer in charge of such quarantine ground and anchorage, or of such quarantine station and anchorage, shall be deemed guilty of a misdemeanor, and upon conviction thereof shall be punished by a fine of not more than three hundred dollars or by imprisonment for not more than one year, or both, in the discretion of the court. That any master or owner of any vessel violating any provision of this act, or any provision of an act entitled "An Act granting additional powers and imposing additional duties on the Marine Hospital Service," approved February fifteenth, eighteen hundred and ninety-three, or violating any rule or regulation made in accordance with this act or said act of February fifteenth, eighteen hundred and ninety-three, relating to the inspection of vessels, or to the prevention of the introduction of contagious or infectious diseases into the United States, or any master, owner, or agent of any vessel making a false statement relative to the sanitary condition of such vessel or its contents, or as to the health of any passenger or person thereon shall be deemed guilty of a misdemeanor, and on conviction thereof shall be punished by a fine of not more than five hundred dollars or imprisonment for not more than one year, or both, in the discretion of the court.

SEC. 5. That in any place where a quarantine station and plant is already established by State or local authorities it shall be the duty of the Secretary of the Treasury, before selecting and designating a quarantine station and grounds and anchorage for vessels, to examine such established stations and plans, with a view of obtaining a transfer of the site and plants to the United States, and whenever the proper authorities shall be ready to transfer the same or surrender the use thereof to the United States, the Secretary of the Treasury is authorized to obtain title thereto or possession and use thereof, and to pay a reasonable compensation therefor, if, in his opinion, such purchase or use will be necessary to the United States for quarantine purposes and the quarantine stations established by authority of this act shall, when so established, be used to prevent the introduction of all quarantinable diseases.

Sec. 6. That whenever any established station, or any land or water, or any part thereof, shall be acquired by the United States under the provisions of this act, jurisdiction over the same shall be ceded to the United States by any State in which the same is situated before any compensation therefor shall be paid.

Sec. 7. That the sum of five hundred thousand dollars, or so much thereof as may be necessary, is hereby appropriated, out of any money in the Treasury not otherwise appropriated, for the purpose of carrying into effect the provisions of this act, as well as for the purpose generally of preventing the importation of yellow fever and other quarantinable diseases into the United States, and for the further purposes, in co-operation with State or municipal health authorities, of eradicating them should they be imported, of preventing their spread from one State into another State, and of destroying their causes.

Approved, June 19, 1906.

